# Wernicke’s Encephalopathy Beyond Alcoholism: A Radio-Clinical Case Series From a Tertiary Care Center in South India

**DOI:** 10.7759/cureus.86954

**Published:** 2025-06-29

**Authors:** Kavitha B Chittaragi, Timothy Chelliah, Sudha Kiran Das, Sidharthan S, Shashank Sharma

**Affiliations:** 1 Department of Radiology, Jagadguru Sri Shivarathreeshwara (JSS) Medical College, JSS Academy of Higher Education and Research, Mysuru, IND; 2 Department of Radiology, Christian Medical College Vellore, Vellore, IND; 3 Department of Radiology, Dhanalakshmi Srinivasan Medical College, Siruvachur, IND

**Keywords:** chronic alcoholism, hyperemesis gravidarum, mri, nonalcoholic we, periaqueductal grey matter, thiamine deficiency, wernicke’s encephalopathy

## Abstract

Introduction

Wernicke’s encephalopathy (WE) is a serious yet potentially reversible neurological condition resulting from thiamine (vitamin B1) deficiency. Although traditionally linked to chronic alcohol consumption, an increasing number of cases are now being recognized in nonalcoholic individuals, where atypical clinical presentations often lead to diagnostic challenges and delays in treatment. This case series highlights a spectrum of WE cases with varied underlying etiologies, along with their clinical manifestations and characteristic neuroimaging findings. By emphasizing the importance of early recognition and imaging, this study aims to increase clinical awareness and promote timely, appropriate management, thereby reducing morbidity and improving outcomes.

Materials and methods

This retrospective case series analyzed 14 patients diagnosed with WE between January 2020 and January 2025 at a tertiary care center in South India. The inclusion criteria were clinical suspicion of WE, with supportive MRI findings showing bilateral symmetrical T2/FLAIR hyperintensities in typical regions (thalami, mammillary bodies, and periaqueductal gray). Patients with alternative diagnoses were excluded. Clinical details, comorbidities, etiological factors, imaging findings, treatment response, and outcomes were reviewed. All patients received thiamine therapy. Descriptive statistics were used to summarize the data.

Results

Among the 14 patients, four were chronic alcoholics, while 10 had nonalcoholic causes such as hyperemesis gravidarum (3), nutritional deficiency (4), parenteral nutrition (2), and CKD on dialysis (1). Most patients presented with altered sensorium and incomplete classical triad symptoms. MRI consistently showed typical WE findings, with additional atypical sites in some nonalcoholic cases. Twelve patients improved with thiamine supplementation, while two were lost to follow-up. All pregnant patients had favorable obstetric outcomes.

Conclusions

WE is often underdiagnosed, especially in nonalcoholic patients, due to atypical presentations. Clinicians and radiologists should maintain a high index of suspicion for WE in patients with risk factors beyond alcohol use. Early diagnosis and treatment are critical to prevent irreversible neurological damage.

## Introduction

Wernicke’s encephalopathy (WE) is an acute, reversible neurological condition caused by a deficiency of thiamine (vitamin B1), characterized classically by the triad of ophthalmoplegia, ataxia, and altered mental status [1]. Although historically associated with chronic alcoholism, nonalcoholic etiologies such as malnutrition, hyperemesis gravidarum, gastrointestinal disorders causing malabsorption, dialysis, and post-surgical states are increasingly recognized as significant contributors to its pathogenesis [2,3]. Diagnosis remains challenging, especially in nonalcoholic individuals, due to atypical or incomplete clinical presentations. MRI is the gold standard imaging modality, revealing characteristic symmetric hyperintensities in the medial thalami, mammillary bodies, and periaqueductal regions. Early recognition and prompt initiation of thiamine therapy are critical to prevent irreversible neurological damage and progression to Korsakoff’s syndrome [3].

## Materials and methods

Study design and setting

This retrospective observational study was conducted at a tertiary care center in South India over five years (January 2020 to January 2025), following approval from the Institutional Ethics Committee of JSS Medical College (approval number: JSSMC/IEC/160525/45 NCT/2024-25).

Inclusion and exclusion criteria

Patients were included based on clinical suspicion of WE and characteristic MRI findings such as bilateral symmetrical hyperintensities on T2/FLAIR involving the thalami, mammillary bodies, or periaqueductal gray. Patients with incomplete records, alternative neurological diagnoses, or suboptimal imaging were excluded from the study.

Data collection, treatment, and outcome

Clinical data, comorbidities, etiologies, MRI features, treatment, and outcomes were collected retrospectively from the hospital records. MRI brain scans were independently reviewed by two experienced radiologists who were blinded to the clinical outcomes. Discrepancies were resolved by consensus. All patients received intravenous thiamine. Outcomes were categorized as recovered, deceased, or lost to follow-up.

Statistical analysis

Data were analyzed descriptively using counts, percentages, and mean values. No inferential statistics were applied due to the limited sample size.

## Results

**Figure 1:**
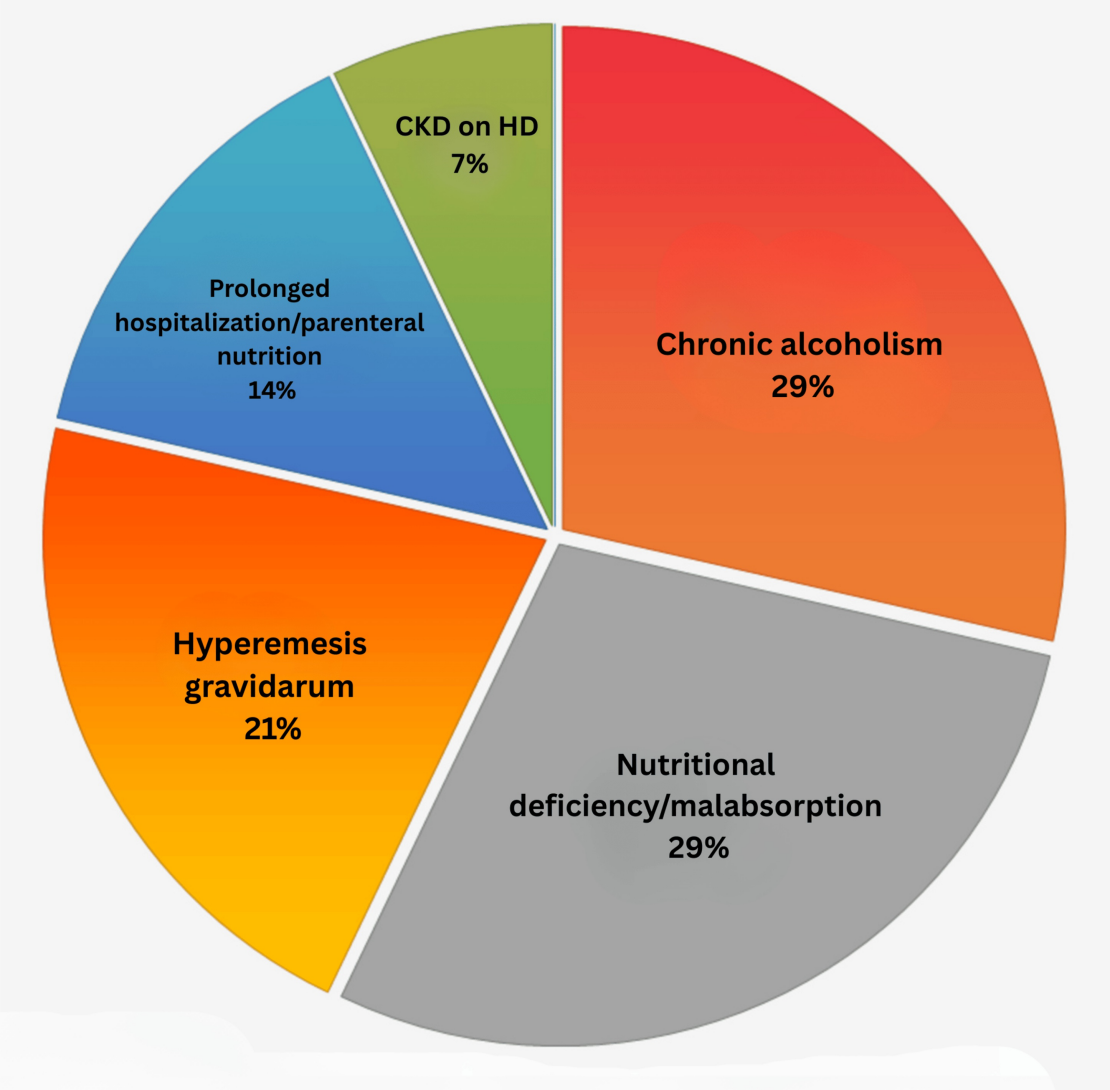
Visual overview of the multifactorial causes of WE (n=14) WE: Wernicke’s encephalopathy, CKD: chronic kidney disease, HD: hemodialysis

This case series includes 14 patients diagnosed with WE at a tertiary care center in South India between 2020 and 2025. The study consisted of nine males and five females, with ages ranging from 24 to 76 years (mean age: 44.8 years). Of the 14 patients, only four had a history of chronic alcohol use, highlighting the growing recognition of nonalcoholic WE in clinical settings. All alcoholic patients were male and exhibited classic clinical and imaging features. In contrast, the remaining 10 patients had nonalcoholic etiologies, including nutritional deficiency or malabsorption (n=4), hyperemesis gravidarum (n=3), prolonged hospitalization with parenteral nutrition (n=2), and end-stage chronic kidney disease on long-term hemodialysis (n=1) (Figure 1).

The clinical symptoms differed among the patients in the group. Altered sensorium was the most frequent symptom, observed in 11 patients, followed by visual disturbances (blurred vision or diplopia) in six patients and limb weakness or gait instability in five patients. Vomiting, either acute or chronic, was noted in four patients, most commonly among pregnant women. The classical triad of global confusion, ophthalmoplegia, and ataxia was often incomplete, particularly in nonalcoholic patients, leading to diagnostic delays. Detailed patient demographics, clinical features, imaging findings, comorbidities, and outcomes are summarized in Table 1.

**Table 1:** Overview of patients demographics, clinical presentation, imaging findings, comorbidities, and outcomes WE: Wernicke's encephalopathy, PTCA: percutaneous transluminal coronary angioplasty, dAVF: dural arteriovenous fistula, ATT: antitubercular therapy, MRI: magnetic resonance imaging

Parameter	Number of patients (%)/value
Demographics	
Total patients	14
Gender (male-to-female ratio)	9:5
Age (range, years)	24-76
Mean age (years)	44.8
Alcoholic WE	4 (28.6%)
Nonalcoholic WE	10 (71.4%)
Nutritional deficiency/malabsorption	4
Hyperemesis gravidarum	3
Parenteral nutrition	2
Chronic kidney disease (on hemodialysis)	1
Clinical and radiological features	
Altered sensorium	11 (78.6%)
Visual disturbances (blurred vision/diplopia)	6 (42.9%)
Limb weakness/gait instability	5 (35.7%)
Vomiting	4 (28.6%)
Classical triad (altered sensorium, ataxia, and ophthalmoplegia)	4 (28.6%)
Imaging findings on MRI (areas involved)	
Medial thalamus	14 (100%)
Mammillary body	13 (92.9%)
Periaqueductal gray matter	12 (85.7%)
Tectal plate	3 (21.4%)
Corpus callosum (Marchiafava-Bignami disease)	1 (7.1%)
Globus pallidus (acquired hepatocerebral degeneration)	1 (7.1%)
Cortical	1 (7.1%)
Comorbidities	
Diabetes mellitus	4 (28.6%)
Hypertension	5 (35.7%)
Ischemic heart disease with PTCA	1 (7.1%)
Pulmonary tuberculosis (on ATT)	1 (7.1%)
dAVF post-embolization	1 (7.1%)
Pregnancy with hyperemesis gravidarum	3 (21.4%)
Outcome	
Recovered	12 (85.7%)
Lost to follow-up	2 (14.3%)
Deceased during follow-up (unrelated to WE)	2 (14.3%)
Healthy term delivery (pregnant women)	3 (100%)

**Figure 2:**
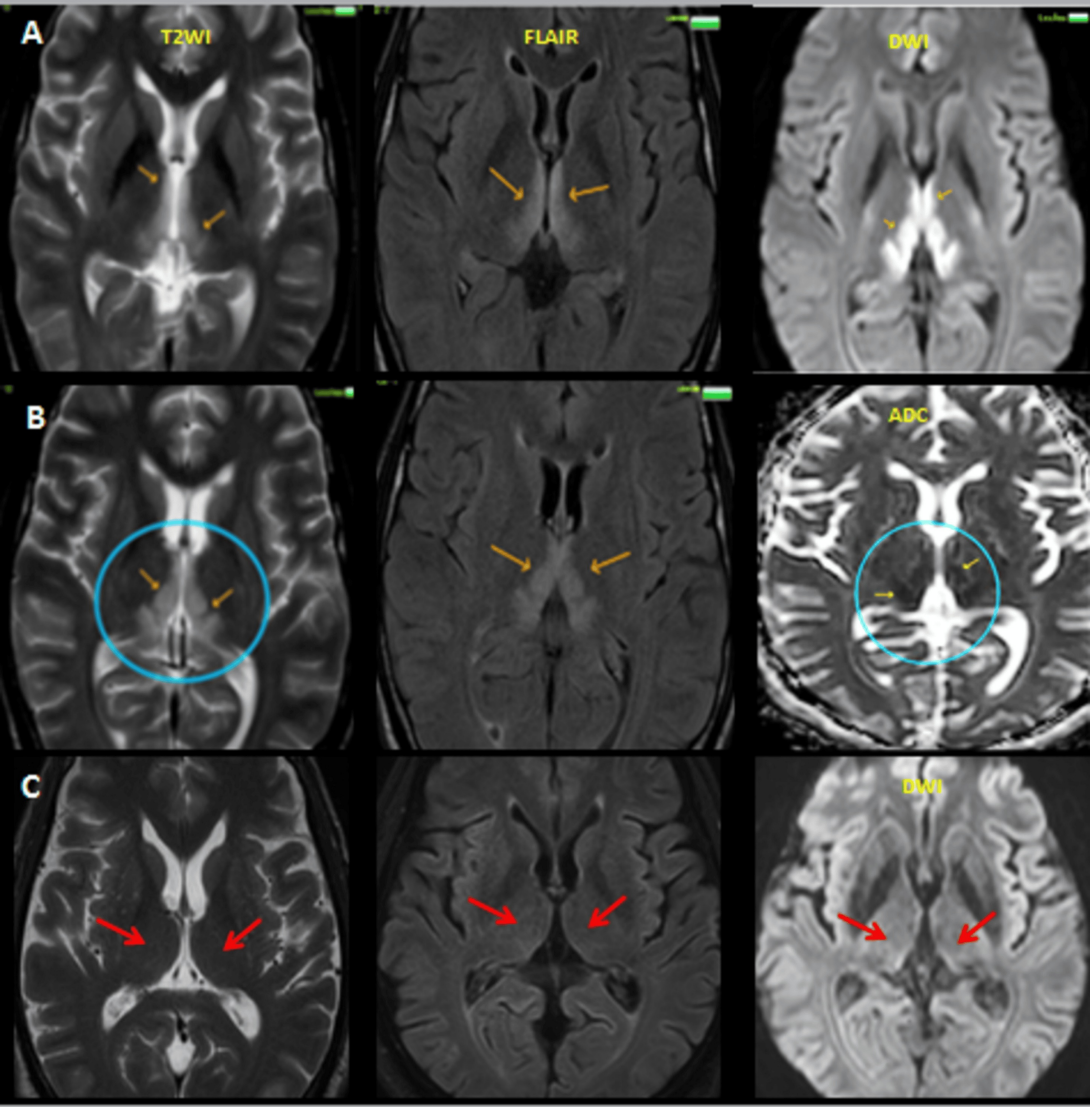
MRI brain of a 58-year-old male who presented with altered sensorium, ataxia, and vomiting following percutaneous transluminal coronary angioplasty for ischemic heart disease. (A & B) Axial T2-weighted, FLAIR, DWI, and ADC images demonstrate symmetrical hyperintensities (orange arrows and circled areas) in the mammillary bodies, dorsomedial thalami, periaqueductal gray matter, and around the third ventricle with diffusion restriction, findings characteristic of acute WE secondary to prolonged parenteral nutrition. (C) Following thiamine supplementation, the patient clinically improved, and follow-up imaging showed resolution of previous abnormalities with normalized signal intensity, indicating radiological recovery (red arrows) MRI: magnetic resonance imaging, FLAIR: fluid attenuated inversion recovery, DWI: diffusion weighted image, ADC: apparent diffusion coefficient, WE: Wernicke’s encephalopathy

Radiological evaluation with MRI consistently demonstrated symmetric T2/FLAIR hyperintensities involving the medial thalami, mammillary bodies, and periaqueductal gray matter (Figure 2).

**Figure 3:**
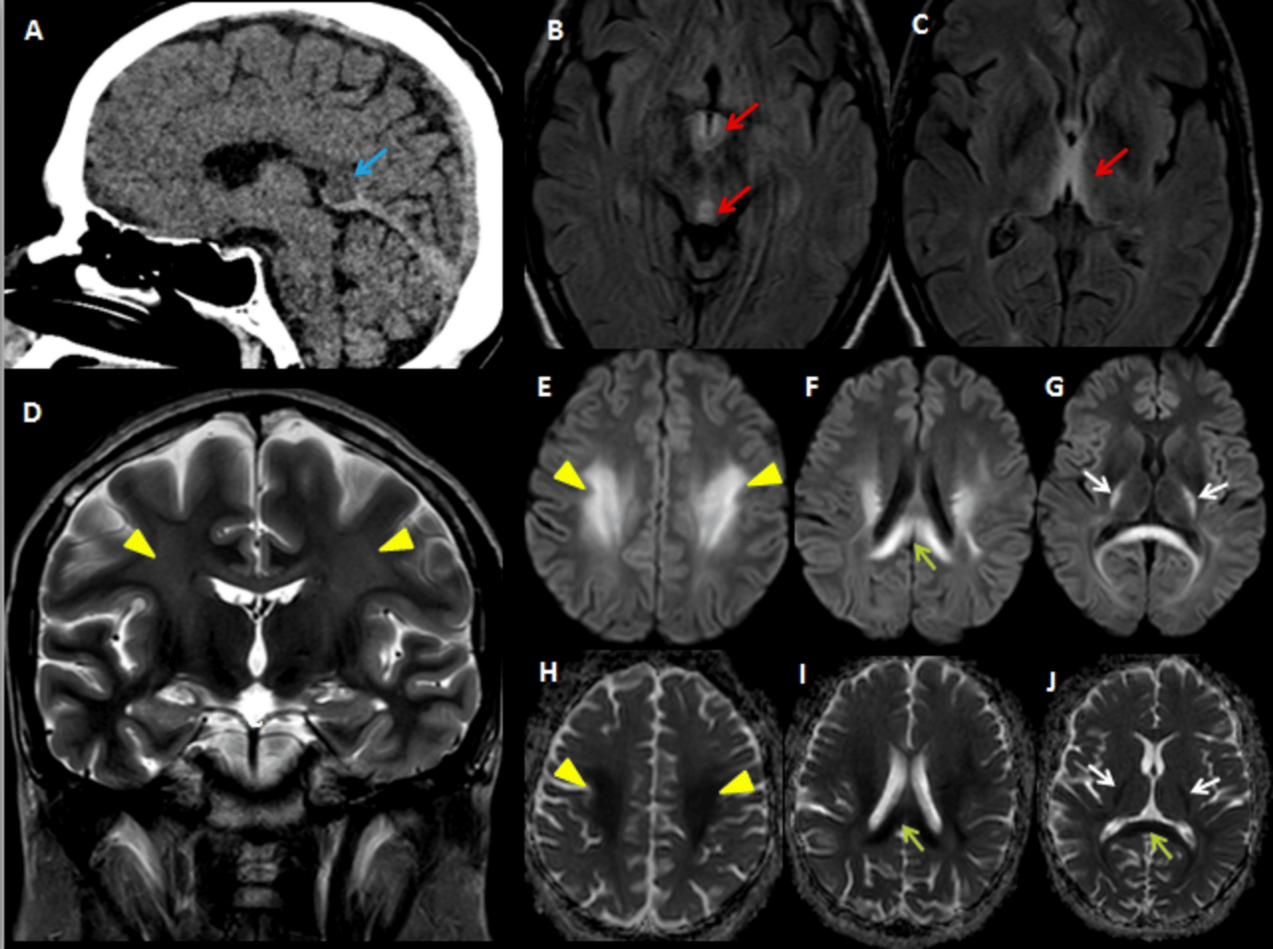
Imaging findings in a 28-year-old male with chronic alcoholism presenting with altered sensorium and paucity of movements. (A) Initial non-contrast CT of the brain shows subtle hypodensity in the splenium of the corpus callosum (blue arrow). (B, C) Further evaluation with MRI, including axial FLAIR sequences, demonstrates symmetric hyperintensities in the dorsomedial thalami and periaqueductal gray matter (red arrows), typical of WE. (D) Coronal T2-weighted image reveals hyperintensities in the bilateral centrum semiovale and corona radiata (yellow arrowheads). (E–G) DWI and (H–J) ADC images show areas of diffusion restriction involving the bilateral centrum semiovale (yellow arrowheads), splenium of the corpus callosum (green arrow), and posterior limb of the internal capsule (white arrows), with corresponding signal drop-out on ADC maps confirming restricted diffusion. These imaging findings are suggestive of a possible overlap between WE and Marchiafava-Bignami disease. The patient was treated with intravenous thiamine and showed symptomatic improvement CT: computed tomography, MRI: magnetic resonance imaging, FLAIR: fluid attenuated inversion recovery, DWI: diffusion weighted image, ADC: apparent diffusion coefficient, WE: Wernicke’s encephalopathy

**Figure 4:**
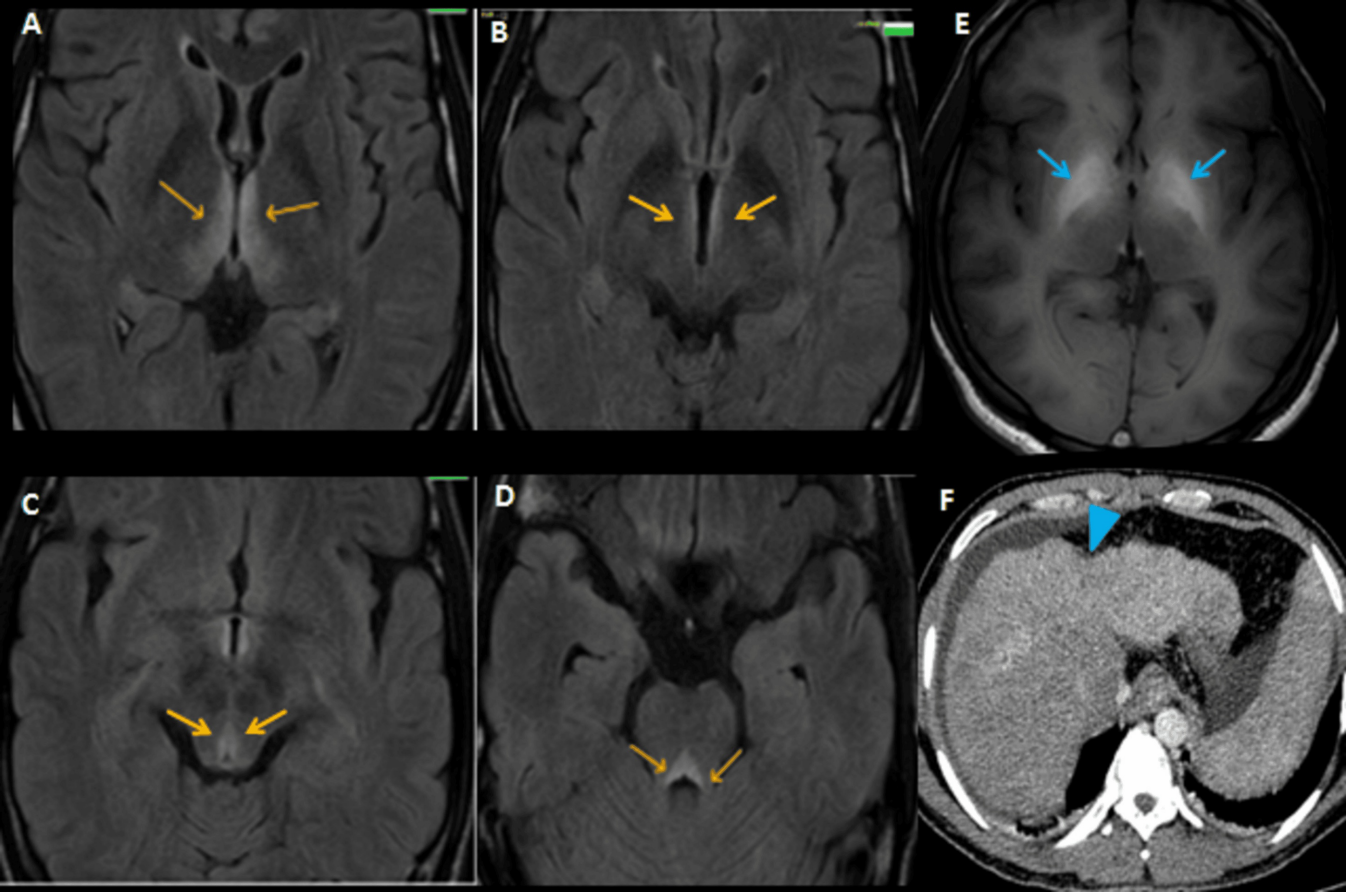
Imaging findings in a 38-year-old male who presented with easy fatigability, abdominal distension, jaundice, slurred speech, and blurred vision. (A–D) MRI and axial FLAIR sequences demonstrate symmetrical hyperintensities around the third ventricle, dorsomedial thalami, and periaqueductal gray matter (orange arrows), consistent with thiamine deficiency-related WE. (E) Axial T1-weighted sequence shows bilateral symmetric hyperintensities in the globus pallidus and subthalamic regions (blue arrows), suggestive of AHCD. (F) To further investigate the underlying etiology, contrast-enhanced CT of the abdomen was performed, which revealed cirrhosis of the liver with portal hypertension (blue arrowhead), confirming alcohol-related liver disease as the likely precipitating factor for the neurological findings CT: computed tomography, MRI: magnetic resonance imaging, FLAIR: fluid attenuated inversion recovery, AHCD: acquired hepatocerebral degeneration, WE: Wernicke’s encephalopathy

Additional findings included tectal plate involvement in three cases, corpus callosum involvement suggestive of Marchiafava-Bignami disease in one case (Figure 3), and globus pallidus involvement with liver cirrhosis indicative of hepatolenticular degeneration in another (Figure 4).

**Figure 5:**
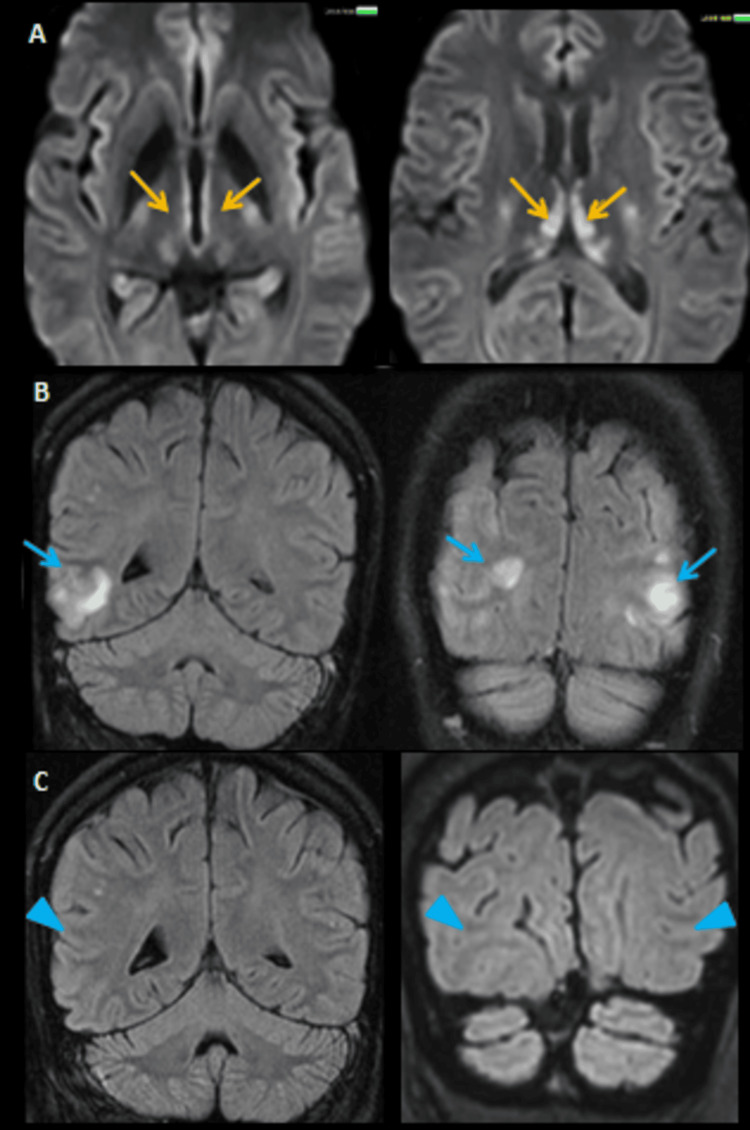
MRI findings in a 76-year-old male with altered sensorium and a history of hypertension, diabetes mellitus, and chronic kidney disease on long-term hemodialysis. (A) Axial DWI sequences show symmetrical hyperintensities around the third ventricle, dorsomedial thalami, and periaqueductal gray matter (orange arrows), suggestive of WE. (B) Axial and coronal FLAIR images reveal bilateral parieto-occipital cortical and subcortical hyperintensities (blue arrows). (C) Follow-up imaging shows resolution of parieto-occipital lobe signal changes on FLAIR sequence, correlating with clinical improvement (blue arrowhead). The imaging features are consistent with dysmetabolic encephalopathy due to a combination of WE and dialysis-related metabolic stress MRI: magnetic resonance imaging, DWI: diffusion-weighted imaging, WE: Wernicke’s encephalopathy, FLAIR: fluid attenuated inversion recovery

Medial thalamic involvement was more prominent in nonalcoholic patients. Cortical involvement was noted in one case (Figure 5).

**Figure 6:**
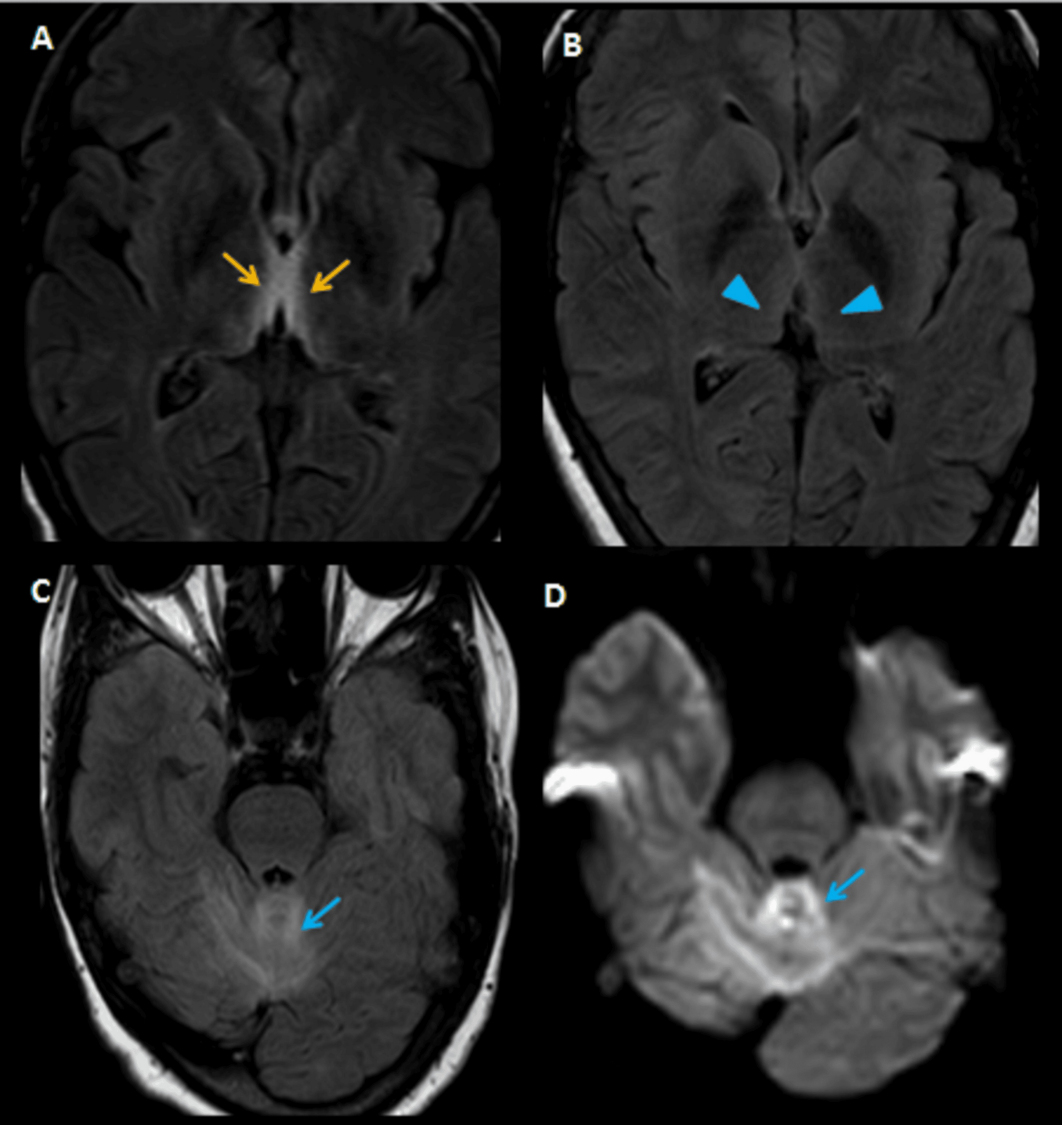
MRI findings in a 25-year-old female with a history of pulmonary tuberculosis and tubercular meningitis on antitubercular therapy, who presented with altered sensorium and blurring of vision. (A) Axial FLAIR image shows symmetrical hyperintensities in the bilateral medial thalami and periaqueductal region (orange arrows), consistent with WE secondary to nutritional deficiency and antitubercular therapy-related malabsorption. (B) Follow-up imaging performed one month later during a subsequent episode of ataxia shows resolution of the previously observed thalamic and periaqueductal hyperintensities (blue arrowhead), indicating radiological improvement of WE. However, new hyperintense signal changes are noted on both FLAIR and DWI sequences in the cerebellum (blue arrows), suggestive of cerebellitis (C&D) MRI: magnetic resonance imaging, FLAIR: fluid attenuated inversion recovery, WE: Wernicke’s encephalopathy, DWI: diffusion-weighted imaging

**Figure 7:**
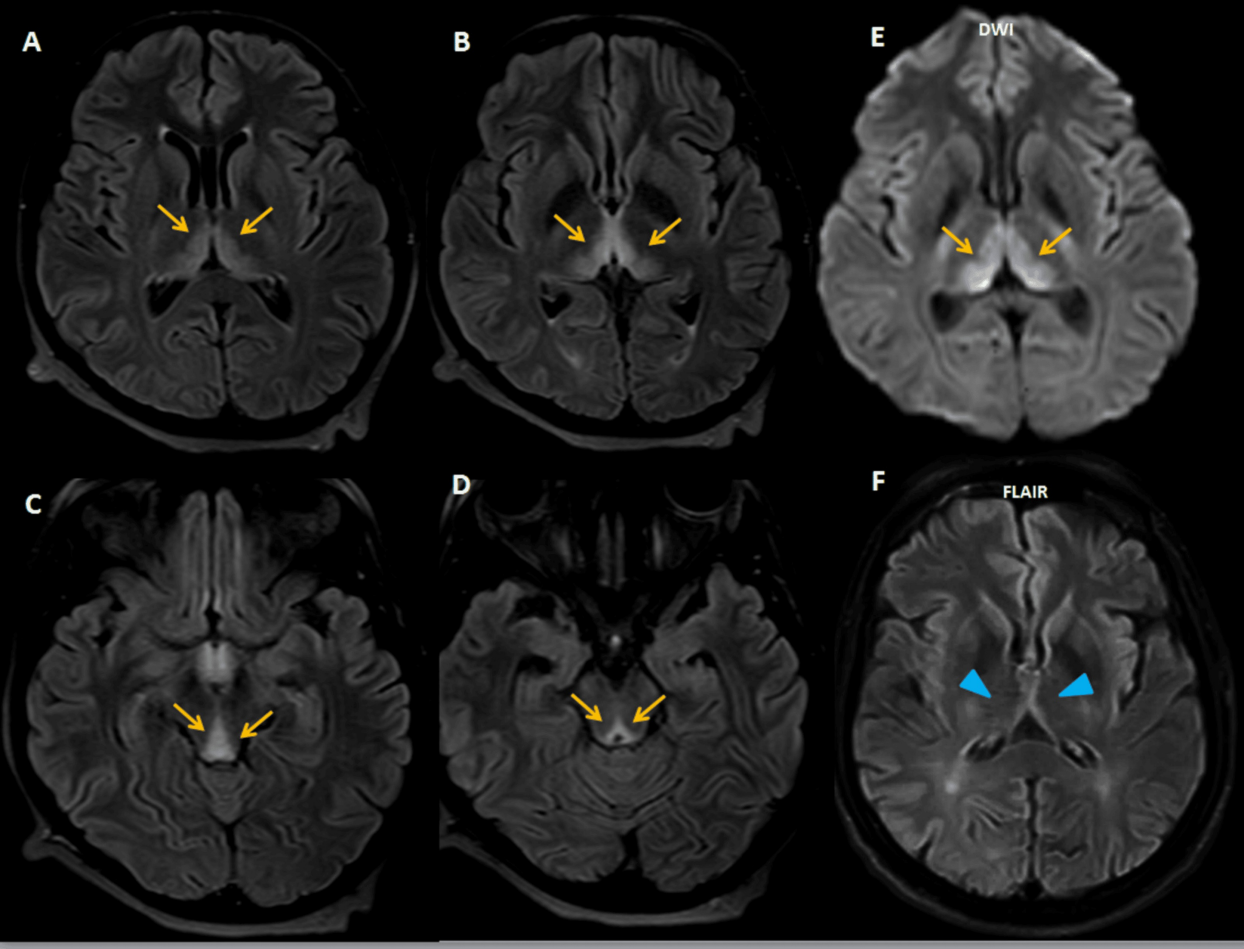
MRI of the brain of a 24-year-old pregnant woman (G3A2, 14 weeks gestation) with a history of persistent vomiting for 15 days presented with limb weakness and slurred speech. (A-D) Axial FLAIR images demonstrate symmetrical hyperintensities (orange arrows) in the dorsomedial thalami, around the third ventricle, periaqueductal gray matter, and tectal plate, typical of acute WE secondary to thiamine depletion resulting from hyperemesis gravidarum. (E) Additional DWI showing corresponding diffusion restriction. (F) Follow-up MRI after thiamine supplementation shows resolution of signal changes, indicating radiological recovery (blue arrowhead). She delivered a healthy baby via elective cesarean at 39 weeks MRI: magnetic resonance imaging, FLAIR: fluid attenuated inversion recovery, WE: Wernicke’s encephalopathy, DWI: diffusion-weighted imaging

Diffusion restriction in the periaqueductal region was a consistent feature across both alcoholic and nonalcoholic groups. Comorbidities were present in the majority of patients, particularly in the nonalcoholic subgroup. Diabetes mellitus was observed in four patients, hypertension in five, and ischemic heart disease in one. One patient was undergoing antitubercular treatment for pulmonary tuberculosis (Figure 6), and three women presented during the first trimester of pregnancy with hyperemesis gravidarum (Figure 7).

These coexisting conditions are thought to exacerbate thiamine deficiency by increasing the body’s metabolic needs, reducing dietary intake, or impairing absorption.

All patients received prompt thiamine replacement therapy, resulting in marked clinical improvement in most cases. Twelve patients achieved full recovery. Two patients were lost to follow-up. Among those who recovered, 10 remained alive and well at the four- to five-year follow-up. The remaining two had initially improved but later died due to causes unrelated to WE, one from sepsis and the other from spontaneous intracranial hemorrhage. Among the pregnant patients, all delivered healthy term infants, with two undergoing elective cesarean sections and one delivering vaginally.

## Discussion

WE was first described by Carl Wernicke in 1881 as “polioencephalitis hemorrhagica superioris” [1]. It has since been recognized as a life-threatening medical emergency requiring prompt diagnosis and intervention. Although traditionally linked to chronic alcoholism, emerging evidence suggests a shifting pattern of WE, with nonalcoholic etiologies accounting for the majority of cases, highlighting the need for clinical suspicion beyond alcoholism [2,3].

The classic triad of ophthalmoplegia, ataxia, and altered mental status is pathognomonic for WE but often incompletely expressed, particularly in nonalcoholic patients. This diagnostic ambiguity necessitates the use of more inclusive clinical criteria, such as those proposed by Caine et al., which suggest the presence of at least two out of four signs: malnutrition, oculomotor abnormalities, cerebellar dysfunction, and confusion for diagnosis [4].

The underlying mechanism of WE is a deficiency of thiamine, a coenzyme essential for cerebral energy metabolism. Thiamine acts as a cofactor for enzymes such as transketolase in the pentose phosphate pathway. Deficiency of which leads to mitochondrial dysfunction, oxidative stress, and eventual neuronal death, particularly in metabolically active regions of the brain [5]. Thiamine deficiency can result from multiple mechanisms, like reduced intake due to malnutrition or hyperemesis gravidarum [6,7], impaired absorption, as seen in gastrointestinal diseases causing malabsorption and post-bariatric surgery [8-10], decreased hepatic storage in chronic liver disease [11], and increased cellular demand or losses, particularly in conditions such as dialysis, malignancy, or refeeding syndrome. Additionally, genetic variations affecting thiamine-utilizing enzymes, such as transketolase, may also predispose certain individuals to develop WE even with minimal thiamine deficiency [12-14]. MRI is the preferred imaging modality for diagnosing WE. Typical findings include bilateral, symmetrical T2/FLAIR hyperintensities involving the mammillary bodies, medial thalami, periaqueductal gray matter, and tectal plate. In addition to the commonly affected regions in WE, several less frequently involved areas may also show abnormal signal intensity on MRI. These include the caudate nucleus, perirolandic cortex, putamina, cranial nerve nuclei, and cortical regions [6,15-17].

In this case series, a substantial number of patients were diagnosed with nonalcoholic WE, with underlying causes such as malnutrition, hyperemesis gravidarum, parenteral nutrition, and chronic kidney disease on dialysis. Regarding clinical presentation, though alcoholic patients often exhibited the classic triad of symptoms, most of the nonalcoholic patients had an incomplete presentation, with more prominent oculomotor disturbances and fewer cerebellar signs. This variation can potentially mislead clinicians and delay diagnosis. Consistent with previous studies, the majority of MRI findings in our series showed symmetric hyperintensities predominantly involving the thalami, mammillary bodies, and periaqueductal regions. Two patients had co-existing Marchiafava-Bignami disease and hepatocerebral degeneration, characterized by altered signal changes in the corpus callosum and T1 hyperintensity in the globus pallidus, respectively. Another patient exhibited cortical involvement, with signal changes in the parieto-occipital lobes (Figure 3). Although cortical involvement is generally associated with poor prognosis, this patient demonstrated both clinical and radiological improvement.

The nonalcoholic causes of WE are diverse and operate through different mechanisms. Malnutrition is one of the most common contributors, either due to reduced intake or increased nutritional demands. Thiamine deficiency results from poor intake and malnutrition, often associated with diets high in processed grains, parenteral nutrition, gastric bypass surgery, malabsorption syndromes, and anorexia nervosa [18]. Netravathi et al. described that hyperemesis gravidarum significantly raises the risk of thiamine deficiency and WE in pregnancy due to poor nutritional intake and increased demands during pregnancy [2]. In a review of 177 cases, 47% of women developed thiamine depletion within six weeks of persistent vomiting, rising to 63% after seven weeks. The lack of thiamine supplementation during this critical period contributed to the onset of WE in many cases, with maternal mortality reported due to delayed recognition and treatment [19]. Several studies have highlighted thiamine deficiency, particularly among hospitalized and critically ill patients. Physiological stress, as well as acute and chronic inflammatory conditions, can deplete thiamine levels. Research also indicates thiamine deficiency in up to 20% of emergency room patients and as high as 70% in those with sepsis. Critically ill ICU patients with lactic acidosis, those on parenteral nutrition, and those undergoing surgery, including coronary artery bypass surgery, are especially vulnerable. In conditions like heart failure, thiamine deficiency often remains undiagnosed, with reported prevalence ranging from 33% to 90% [20-22]. Hung et al. concluded that patients undergoing regular hemodialysis for end-stage kidney disease are at increased risk of thiamine deficiency-related encephalopathy, which can clinically resemble uremic encephalopathy but typically responds well to thiamine supplementation [14]. Comorbidities such as diabetes are linked to lower thiamine levels, primarily due to hyperglycemia-induced impaired cellular uptake and increased urinary excretion, which can predispose these patients to developing WE [23]. These factors underscore the importance of careful monitoring and routine multivitamin supplementation to minimize the risk of developing complications, such as WE.

Despite being a treatable condition, delayed diagnosis of WE can lead to irreversible complications such as Korsakoff’s psychosis. Timely parenteral thiamine administration remains the cornerstone of therapy, with guidelines recommending 200-500 mg IV/oral thiamine BD or TID daily, depending on severity, until recovery. An alternative approach during an acute crisis is to administer 50 mg intramuscularly for two to four days, followed by oral maintenance therapy [18,24]. While ocular symptoms often improve rapidly, persistent gait disturbances and cognitive impairment are common, especially in alcohol-related WE. Educating patients about the link between alcohol misuse and thiamine deficiency is crucial, alongside implementing a structured substance misuse treatment plan. For nonalcoholic causes, it is important to manage the underlying condition, maintain adequate long-term nutritional support, and offer prophylactic thiamine supplementation to at-risk individuals to prevent the occurrence.

This study has certain limitations, including its retrospective design and the small sample size from a single tertiary care center, which may limit the generalizability of the findings. Additionally, variability in thiamine dosing and the absence of standardized long-term neurological follow-up may have influenced outcome assessment. Incomplete documentation in medical records may also have introduced some degree of reporting bias. Future research should focus on developing more sensitive biomarkers for early diagnosis, evaluating the efficacy of various thiamine dosing regimens, and exploring genetic susceptibility to thiamine metabolism disorders.

## Conclusions

WE, though classically associated with chronic alcoholism, is increasingly being recognized in nonalcoholic patients, where diagnosis is often delayed due to atypical clinical presentations. These findings emphasize the critical importance of clinician awareness and radiologist vigilance in diagnosing WE beyond alcoholism. A strong clinico-radiological correlation, particularly through MRI, facilitates early diagnosis and timely initiation of thiamine therapy, leading to significant recovery in most cases without neurological deficits. Nutritional supplementation should be prioritized for individuals with suspected deficiencies or at risk of malabsorption. Prophylactic thiamine administration is especially important in high-risk groups such as hospitalized patients receiving prolonged parenteral nutrition or undergoing dialysis to effectively prevent the onset of WE.
